# Immune checkpoint inhibitor-related thrombocytopenia: incidence, risk factors and effect on survival

**DOI:** 10.1007/s00262-021-03068-2

**Published:** 2021-10-07

**Authors:** Tyler C. Haddad, Songzhu Zhao, Mingjia Li, Sandip H. Patel, Andrew Johns, Madison Grogan, Gabriella Lopez, Abdul Miah, Lai Wei, Gabriel Tinoco, Brian Riesenberg, Zihai Li, Alexa Meara, Erin M. Bertino, Kari Kendra, Gregory Otterson, Carolyn J. Presley, Dwight H. Owen

**Affiliations:** 1 Division of Medical Oncology, Department of Internal Medicine , The Ohio State University - James Comprehensive Cancer Center, 1800 Cannon Drive, Suite 1335, Columbus, OH 43210 USA; 2 Center for Biostatistics, The Ohio State University - James Comprehensive Cancer Center, 1800 Cannon Drive, Suite 1335, Columbus, OH 43210 USA; 3 Division of Rheumatology and Immunology, The Ohio State University - James Comprehensive Cancer Center, 1800 Cannon Drive, Suite 1335, Columbus, OH 43210 USA

**Keywords:** Immune checkpoint inhibitor (ICI), Immunotherapy, Thrombocytopenia, Immune-related adverse events (irAE)

## Abstract

**Introduction:**

Immune checkpoint inhibitors (ICI) are associated with unique immune-related adverse events (irAEs). Immune-related thrombocytopenia (irTCP) is an understudied and poorly understood toxicity; little data are available regarding either risk of irTCP or the effect of irTCP on clinical outcomes of patients treated with ICI.

**Methods:**

We conducted a retrospective review of sequential cancer patients treated with ICI between 2011 and 2017 at our institution. All patients who received ICI alone or in combination with other systemic therapy in any line of treatment were included; those with thrombocytopenia ≥ grade 3 at baseline were excluded. We calculated the incidence of ≥ grade 3 irTCP and overall survival (OS). Patient factors associated with irTCP were assessed.

**Results:**

We identified 1,038 patients that met eligibility criteria. Overall, 89 (8.6%) patients developed grade ≥ 3 thrombocytopenia; eighteen were attributed to ICI (1.73% overall). Patients who developed grade ≥ 3 irTCP had worse overall survival compared to those whose thrombocytopenia was unrelated to ICI (4.17 vs. 10.8 month; HR. 1.94, 95% CI 1.13, 3.33; log-rank *p* = 0.0164). Patients with grade ≥ 3 irTCP also had worse survival compared to those without thrombocytopenia (4.17 vs. 13.31 months; HR 2.22, 95% CI 1.36, 3.62; log-rank *p* = 0.001). The incidence of irTCP appeared lowest among those treated with PD-1/L1 monotherapy (*p* = 0.059) and was not associated with cancer type, smoking status, age, gender, race, or line of therapy.

**Conclusions:**

Unlike other irAEs, we found that irTCP was associated with worse overall survival. The incidence of irTCP appeared lowest among those treated with PD-1/L1 monotherapy.

**Supplementary Information:**

The online version contains supplementary material available at 10.1007/s00262-021-03068-2.

## Introduction

Immune checkpoint inhibitors (ICI) are a standard of care therapy for patients with many different cancers. ICI are generally well tolerated but are associated with a unique set of toxicities termed immune-related adverse events (irAE) [1]. These can affect any organ system but most commonly include colitis, pneumonitis, dermatitis, and endocrine dysfunction; the toxicities are generally reversible but can cause morbidity and mortality, and can impact the ability to deliver ongoing therapy [2–4]. Thrombocytopenia is an uncommon and therefore poorly characterized irAE; severe cases can even be life-threatening [5]. Severe cases of ICI-related immune thrombocytopenia have been reported among patients with non-small cell lung cancer (NSCLC), usually associated with having elevated platelet-associated immunoglobulin indicating an immune-driven mechanism, as well as in melanoma and renal cell carcinoma [6–9]. ICI-related thrombotic thrombocytopenic purpura (TTP) has been found to occur rarely in each of these cancer types [10–12].

Thrombocytopenia can occur in patients with advanced cancer due to a number of reasons beyond ICI, including sepsis, antibiotics and other medications, liver failure, and disseminated intravascular coagulation of malignancy [13–19]. This may lead to missed diagnosis and thus under approximation of its true incidence. In the few studies that have attempted to describe the incidence of immune-related thrombocytopenia (irTCP), estimates range from 0.2 to 2.8% [8, 20–22]. Although several studies have found an association between irAE and clinical benefit, most studies have not evaluated hematologic toxicities generally or thrombocytopenia specifically in this context. [23–25] At least one prior study has looked at the association of thrombocytopenia of any etiology during treatment with ICI and overall survival (OS) [26]. However, to our knowledge there have been no studies to date evaluating whether irTCP has an effect on clinical outcomes including survival. We therefore conducted a study to evaluate the patterns of incidence of serious irTCP including an assessment of risk factors, and evaluate whether irTCP is associated with OS.

## Methods

We conducted a retrospective review of sequential patients with any solid or hematologic cancer treated with checkpoint inhibitors between January 2011 and June 2017 at The Ohio State University Comprehensive Cancer Center. Eligible patients received at least one dose of an immune checkpoint inhibitor in any line of treatment for advanced cancer as part of standard of care or as a participant in a clinical trial. Checkpoint inhibitors included monoclonal antibodies to PD-1, PD-L1, or CTLA-4 alone or in combination therapies. Baseline patient characteristics were assessed from the physician clinical care documentation and electronic medical record. Research Electronic Data Capture (REDCap) was used for data collection [27]. Patients were assessed for baseline platelet count and lowest platelet count occurring after the first dose of ICI. Thrombocytopenia grading was performed utilizing the Common Terminology Criteria for Adverse Events, version 5.0; patients with grade 3 or 4 thrombocytopenia at baseline were excluded. Cases with grade 3 (25,000–50,000 plt/mcL) or 4 (< 50,000 plt/mcL) thrombocytopenia occurring after ICI were manually reviewed for attribution to ICI therapy compared to other causes. Attribution was based on association of thrombocytopenia with ICI use, lack of other clear risk factors, or improvement with holding ICI or treatment with immune suppressive therapies. The institutional review board of The Ohio State University Medical Center approved this study (#2016C0070).

### Statistical analysis

Patient characteristics were summarized using descriptive statistics including medians and interquartile ranges for the continuous variables and frequencies for the categorical variables for all the patients, as well as for patients with or without grade 3 or higher thrombocytopenia, respectively. Kruskal–Wallis test was used in the analysis of determining patient factors associated with at least grade 3 thrombocytopenia for continuous variables and Fisher's exact test was used for categorical variables. Among patients with grade 3 or higher thrombocytopenia, patient characteristics have been compared between Likely irTCP and Unlikely irTCP using Fisher's exact test for the categorical variables and Kruskal Wallis test for the continuous variables. OS was calculated from the date of initiation of the ICI to death from any cause or date of the last follow-up. Median overall survival with 95% confidence intervals was estimated using the Kaplan–Meier method. Log-rank test was used to compare survival curves. Cox proportional hazard models were used to examine the univariate associations between platelet categories with overall survival. *P* values < 0.05 were considered statistically significant. All analyses were conducted in SAS version 9.4 (SAS Institute, Cary, North Carolina).

## Results

### Patient characteristics

We identified 1038 patients meeting criteria for this study. Detailed patient characteristics are listed in Table 1. The median age was 61.4 (range 19.4, 92). The study population included patients with a variety of cancer types and lines of therapy. The most common ICIs used were PD-1/PD-L1 inhibitors (*n* = 730, 70.2%), and patients were treated across multiple lines of therapy (first line, *N* = 369, 36%). Median OS of the entire cohort was 11.93 months with a median follow-up was of 11.89 months.

**Table 1:** Patient characteristics

Variable	Description	Total (*n* = 1038)
Age	Median [IQR] (min, max)	61.4 [53.8, 69.1] (19.4, 92)
Race	White	960 (92.5%)
	Non-white	78 (7.5%)
Sex	Male	613 (59.1%)
	Female	425 (40.9%)
Smoking status	No	425 (40.9%)
	Yes	613 (59.1%)
History of HTN	No	754 (72.6%)
	Yes	284 (27.4%)
History of DM	No	832 (80.2%)
	Yes	206 (19.8%)
Type of immunotherapy	PD-1/L1	730 (70.3%)
	CTLA-4	194 (18.7%)
	Combination PD-1/CTLA-4	77 (7.4%)
	Other	37 (3.6%)
Cancer type	Melanoma	337 (32.5%)
	NSCLC + SCLC	213 (20.5%)
	RCC	114 (11%)
	Head and neck	64 (6.2%)
	Other	310 (29.9%)
BMI	Median [IQR] (min, max)	Missing = 16 27.6 [23.6, 31.7] (13.4, 70.2)
Line of therapy	Missing	18
	1	369 (36.2%)
	2	301 (29.5%)
	> = 3	350 (34.3%)

### Incidence and risk factors for grade 3 or higher thrombocytopenia of any etiology

Of 1038 patients treated with ICI, 89 (8.6%) developed grade 3 or 4 thrombocytopenia, whereas 328 (31.6%) developed grade 1 thrombocytopenia and 65 (6.3%) developed grade 2 thrombocytopenia. The median (interquartile) time to grade 3 thrombocytopenia was 72.5 (30.5, 153) days. We evaluated risk factors for development of grade 3 or higher thrombocytopenia including age, race, gender, smoking history, type of immunotherapy, cancer type, BMI and line of therapy. Smoking status (*p* = 0.024), cancer type (*p* < 0.001) and BMI (*p* = 0.01) were significantly associated with development of grade 3 of higher thrombocytopenia of any etiology (Table 2).

**Table 2:** Differences in patient characteristics according to whether grade 3 or higher thrombocytopenia occurred

Variable	Description	1:Grade 0–2 toxicities (*n* = 949)	2:Grade 3–4 toxicities (*n* = 89)	Total (*n* = 1038)	*p*-value
Age	Median [IQR] (min, max)	Missing = 0 61.8 [54.2, 69.1] (19.4, 92)	Missing = 0 59.7 [52.5, 68] (19.4, 85.1)	missing = 0 61.4 [53.8, 69.1] (19.4, 92)	0.0903
Race	White	881 (92.8%)	79 (88.8%)	960 (92.5%)	0.2023
	Non-white	68 (7.2%)	10 (11.2%)	78 (7.5%)	
Sex	Male	562 (59.2%)	51 (57.3%)	613 (59.1%)	0.7363
	Female	387 (40.8%)	38 (42.7%)	425 (40.9%)	
Smoking status	No	378 (39.8%)	47 (52.8%)	425 (40.9%)	0.0237
	Yes	571 (60.2%)	42 (47.2%)	613 (59.1%)	
Type of immunotherapy	PD-1/L1	668 (70.4%)	62 (69.7%)	730 (70.3%)	0.3949
	CTLA-4	181 (19.1%)	13 (14.6%)	194 (18.7%)	
	Combination PD-1/CTLA-4	68 (7.2%)	9 (10.1%)	77 (7.4%)	
	Other	32 (3.4%)	5 (6.7%)	37 (3.6%)	
Cancer type	Melanoma	314 (33.1%)	23 (25.8%)	337 (32.5%)	0.0003
	NSCLC + SCLC	198 (20.9%)	15 (16.9%)	213 (20.5%)	
	RCC	111 (11.7%)	3 (3.4%)	114 (11%)	
	Head and neck	61 (6.4%)	3 (3.4%)	64 (6.2%)	
	Other	265 (27.9%)	45 (50.6%)	310 (29.9%)	
BMI	Median [IQR] (min, max)	Missing = 14 27.5 [23.4, 31.6] (13.4, 70.2)	Missing = 2 29.1 [24.9, 33.5] (17, 50.2)	Missing = 16 27.6 [23.6, 31.7] (13.4, 70.2)	0.0107
Line of therapy	Missing	17	1	18	0.0789
	1	342 (36.7%)	27 (30.7%)	369 (36.2%)	
	2	280 (30%)	21 (23.9%)	301 (29.5%)	
	> = 3	310 (33.3%)	40 (45.5%)	350 (34.3%)	

### Incidence and risk factors for grade 3 or higher immune-related thrombocytopenia

Of the 89 patients who developed at least grade 3 thrombocytopenia, 18 (20%) were attributed to ICI after manual review and exclusion of other etiologies (1.73% of entire cohort). We evaluated risk factors for irTCP including age, race, gender, smoking history, type of immunotherapy, cancer type, BMI and line of therapy; none were significantly associated with irTCP (Table 3), however incidence of irTCP appeared lowest among those treated with PD-1/L1 monotherapy (*p* = 0.059). For those who developed grade 3 or higher irTCP, five had grade 1 thrombocytopenia and three had grade 2 thrombocytopenia prior to ICI therapy. Patient-specific details are listed in Supplementary Table 1.

**Table 3:** Differences in patient characteristics who developed grade 3 or higher thrombocytopenia according to whether toxicity was immune-related

Variable	Description	1:Likely/possible immune toxicity (*n* = 18)	2:Unlikely immune toxicity (*n* = 71)	Total (*n* = 89)	*p*-value
Age	Median [IQR] (min, max)	57.8 [50, 68] (35.7, 77.1)	60.1 [52.5, 69.2] (19.4, 85.1)	59.7 [52.5, 68] (19.4, 85.1)	0.7671
Race	White	18 (100%)	61 (85.9%)	79 (88.8%)	0.2029
	Non-white	0 (0%)	10 (14.1%)	10 (11.2%)	
Sex	Male	10 (55.6%)	41 (57.7%)	51 (57.3%)	1.0000
	Female	8 (44.4%)	30 (42.3%)	38 (42.7%)	
Smoking status	No	12 (66.7%)	35 (49.3%)	47 (52.8%)	0.2904
	Yes	6 (33.3%)	36 (50.7%)	42 (47.2%)	
Type of immunotherapy	PD-1/L1	8 (44.4%)	54 (76.1%)	62 (69.7%)	0.0591
	CTLA-4	4 (22.2%)	9 (12.7%)	13 (14.6%)	
	Combination PD-1/CTLA-4	4 (22.2%)	5 (7%)	9 (10.1%)	
	Other	2 (11.1%)*	3 (4.2%)^†^	5 (5.6%)	
Cancer type	Melanoma	9 (50%)	14 (19.7%)	23 (25.8%)	0.0752
	NSCLC + SCLC	1 (5.6%)	14 (19.7%)	15 (16.9%)	
	RCC	0 (0%)	3 (4.2%)	3 (3.4%)	
	Head and neck	1 (5.6%)	2 (2.8%)	3 (3.4%)	
	Other	7 (38.9%)	38 (53.5%)	45 (50.6%)	
BMI	Median [IQR] (min, max)	missing = 0 28.9 [24.9, 32.3] (17, 47.1)	missing = 2 29.1 [25.6, 33.8] (19.1, 50.2)	missing = 2 29.1 [24.9, 33.5] (17, 50.2)	0.7060
Line of therapy	Missing	1	0	1	0.9394
	1	6 (35.3%)	21 (29.6%)	27 (30.7%)	
	2	4 (23.5%)	17 (23.9%)	21 (23.9%)	
	> = 3	7 (41.2%)	33 (46.5%)	40 (45.5%)	

^*^Pembrolizumab + acalabrutinib (*n* = 2). ^†^Pembrolizumab + BMS-986156, a glucocorticoid-induced tumor necrosis factor receptor-related protein antagonist monoclonal antibody (*n* = 1), pembrolizumab + acalabrutinib (*n* = 1), nivolumab + cisplatin (*n* = 1)

### Bleeding complications, treatment, and response for irTCP

Four patients had bleeding complications related to irTCP. Two suffered gastrointestinal bleed, including one grade 5 event (death). One patient had a subdural hematoma and one had hemorrhagic conversion of a brain metastasis.

Seven patients received treatment for irTCP. Two patients received treatment with oral steroids alone (prednisone *n* = 1 and dexamethasone *n* = 1) without experiencing improvement in thrombocytopenia. Two patients received treatment with IVIG including one patient in combination with romiplostim and one patient in combination with prednisone; neither patient had a treatment response. One patient was treated with oral prednisone followed by intravenous methylprednisolone without improvement.

Two patients that were treated for irTCP had a response. The first received treatment with oral prednisone, oral dexamethasone, and eltrombopag. He began treatment with nivolumab for metastatic melanoma with a platelet count of 80,000 plt/mcL. By cycle 3 day 2 (58 days) his platelet count fell to 52,000 plt/mcL. He received platelet transfusions but improvement in thrombocytopenia was not sustained. He was treated with high dose oral prednisone starting on cycle 3 day 12 with improvement from 58,000 to 93,000 plt/mcL five days later. He was transitioned to an oral dexamethasone taper. Thrombocytopenia worsened and ultimately his platelet count fell to 22,000 plt/mcL despite transitioning back to high dose oral prednisone. He was treated with eltrombopag with improvement to 95,000 plt/mcL and he restarted nivolumab therapy without recurrence of thrombocytopenia. His platelet count peaked at 231,000 plt/mcL 29 days after starting eltrombopag. The second patient received oral prednisone followed by intravenous methylprednisolone. She began treatment for non-small cell lung cancer with pembrolizumab with a platelet count of 202,000 plt/mcL. At cycle 2 day 22 she was found to have grade 3 transaminitis felt to represent autoimmune hepatitis; treatment with pembrolizumab was discontinued at that time and on that date her platelet count was 134,000 plt/mcL. She was treated with high dose oral prednisone for autoimmune hepatitis and during that time her platelet count ultimately fell to 23,000 plt/mcL, eighty-one days after starting pembrolizumab. Treatment was switched to intravenous methylprednisolone and thrombocytopenia improved with a final platelet count of 75,000 plt/mcL sixteen days later, at which time the patient transitioned to hospice care due to progressive lung cancer.

### Effect of thrombocytopenia on OS

**Fig. 1:**
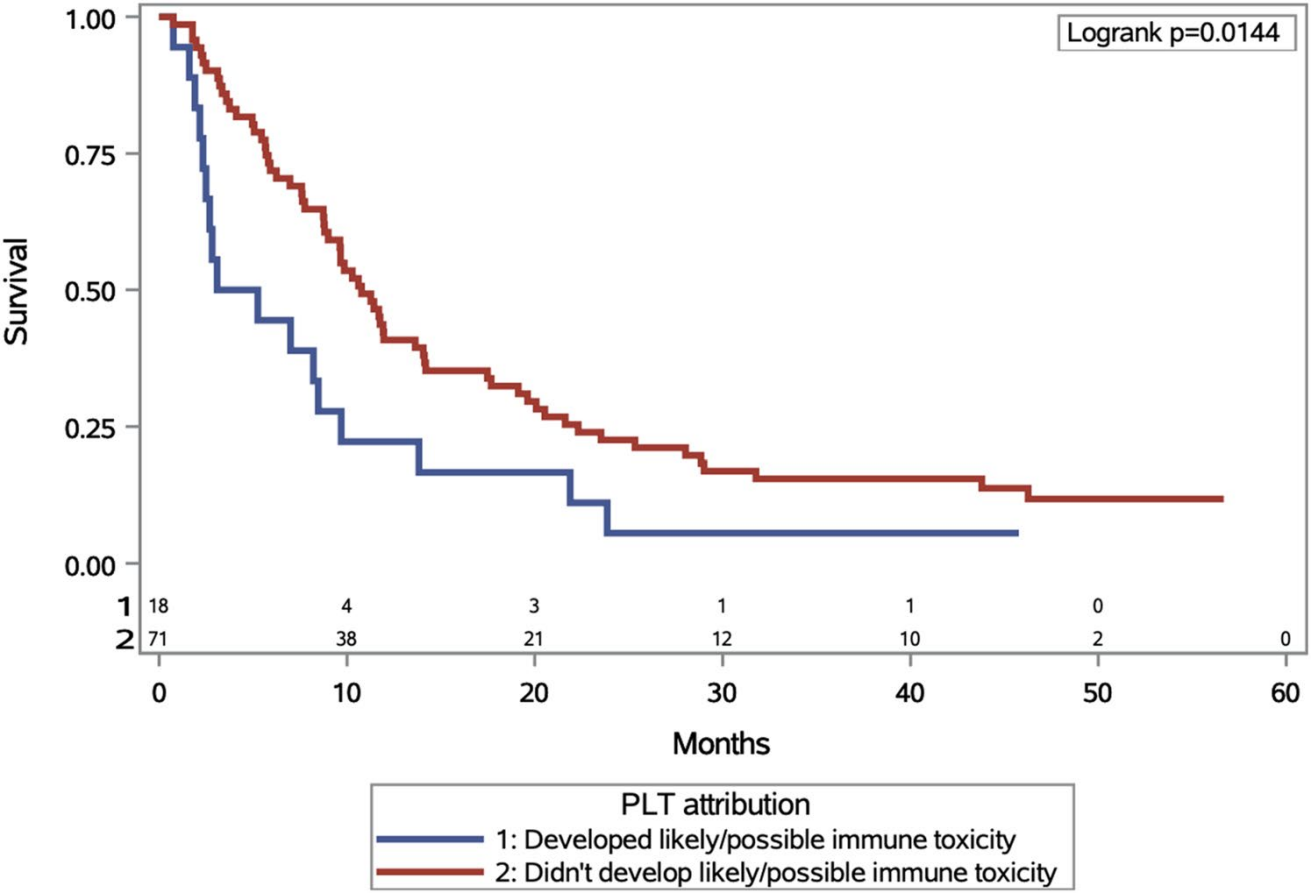
Kaplan—Meier analysis OS among those who developed at least grade 3 thrombocytopenia according to whether toxicity was immune-related. OS, overall survival

**Fig. 2:**
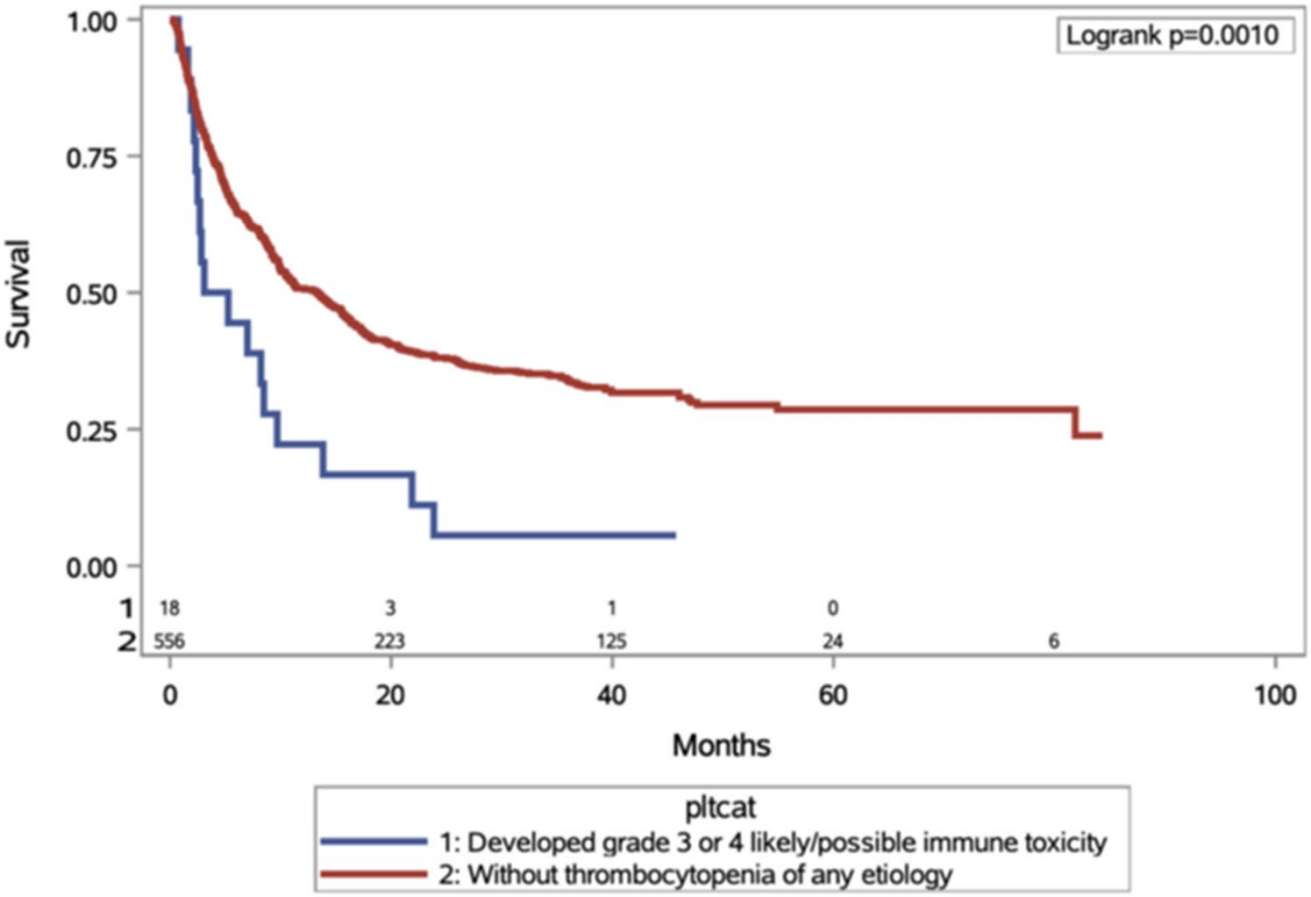
Kaplan—Meier analysis OS among those who developed at least grade 3 immune thrombocytopenia versus those without thrombocytopenia of any etiology. OS, overall survival

Patients who developed grade 3 or higher irTCP had worse overall survival compared to those whose thrombocytopenia was unrelated to ICI (4.17 vs. 10.8 month; HR. 1.94, 95% CI 1.13, 3.33 log-rank *p* = 0.0164) as shown in Fig. 1. Patients with grade 3 or higher irTCP also had worse overall survival compared to those without thrombocytopenia of any etiology (4.17 vs. 13.31 months; HR 2.22, 95% CI 1.36, 3.62; log-rank *p* = 0.001) as shown in Fig. 2. Sixteen patients died from disease progression, one from acute hypoxic respiratory failure of unknown etiology, one from gastrointestinal bleed, one from sepsis, and one is still living.

## Discussion

In this study population, we found that the incidence of grade 3 or higher immune-related thrombocytopenia was 1.73%, and that patients who experienced immune-related thrombocytopenia had worse overall survival. To our knowledge, this is the first study to find an association between an irTCP and worse clinical outcomes in patients treated with ICI. Given that prior studies have demonstrated an association between the development of irAE and clinical benefit, the finding that irTCP was associated with worse survival is of high clinical relevance and warrants further exploration. The reason for this finding is unclear, although there are several possibilities including that the mechanism of action of irTCP may differ than that of other irAE such as pneumonitis or hypothyroidism, that irTCP may be associated with increased significant bleeding events, that the occurrence of irTCP may limit subsequent therapeutic options such as cytotoxic chemotherapy, or that the treatment of irTCP may itself be associated with higher risk for poor outcomes.

One major challenge in studying irTCP in this patient population is that thrombocytopenia may occur due to a variety of common exposures, medications, and comorbid conditions that influence platelet count. Past and concurrent chemotherapy is a major cause of mild to life-threatening thrombocytopenia [28–30]. Those with cancer are at increased risk of a variety of viral and bacterial infections, many of which are associated with both decreased platelet production and enhanced destruction [31–34]. Patients often receive antimicrobial medications that can further contribute to thrombocytopenia including vancomycin, penicillins, linezolid, trimethoprim-sulfamethoxazole, and others [13–15]. Hospitalization is common and increases exposure to heparin, thus increasing the risk of heparin-induced thrombocytopenia [35]. Moreover, thrombocytopenia is often encountered among critically ill patients and has a variety of mechanisms, including sepsis-mediated etiologies and disseminated intravascular coagulation [16, 36]. Thrombocytopenia is also a sequela of medical diseases such as chronic liver disease due to decreased production of thrombopoietin and splenic sequestration in the setting of portal hypertension, and also in acute liver failure although the mechanism is less understood [17, 18]. For this reason, we utilized specific criteria to aid in the diagnosis of immune-related adverse events. These include association of thrombocytopenia with ICI use, lack of other clear risk factors, or improvement with holding ICI or treatment with immune suppressive therapies. These criteria should be borne in mind when encountering patients treated with ICI who develop thrombocytopenia. Given the numerous reasons for thrombocytopenia in this patient population, it was felt that excluding cases with any level of baseline thrombocytopenia would severely restrict the study population and would create bias. However, since the primary endpoint of this study was the incidence of grade 3 higher irTCP, those with baseline grade 3 or 4 thrombocytopenia were excluded.

The largest recent study that described irTCP was by Kramer et al., who reviewed 7,626 patients treated with ICI from sixteen cancer centers and found that 0.2% developed thrombocytopenia which was probably or certainly related to treatment. Of these, 92% had melanoma, cutaneous squamous cell carcinoma, or merkel cell carcinoma; however, the cancer types of the overall screened population were not described. Given the challenge of identifying thrombocytopenia as immune-related in this patient population, excluding cases that are uncertain to be related to ICI may underestimate the true incidence. Le Burel et al., who retrospectively reviewed 908 patients with any cancer type treated with anti-PD-1/PD-L1 also found that 0.2% developed irTCP [20]. It is unclear whether those that were possibly or probably related to ICI were included in the estimate. Petrelli et al. performed a systematic review and meta-analysis of forty-seven trials including 9,324 patients with solid tumors treated with PD-L1 inhibitor monotherapy or in combination immunotherapy for incidence of hematologic irAEs; however, this study primarily focused on anemia. For the purposes of evaluating thrombocytopenia, only thirteen trials were included and the incidence of all-grade thrombocytopenia was 2.84% and grade 3–5 was 1.83% [21]. This study did not specifically state how many patients were included in the subset analysis of the thirteen trials, excluded patients with hematologic malignancies, and did not clarify whether attribution of thrombocytopenia was to immunotherapy. Shiuan et al. reviewed 2,360 patients with melanoma treated with ICI and identified eleven cases (0.47%) that developed irTCP [8]. It is notable that in this study, we found the incidence of irTCP was lower among melanoma patients (0.30%) than the total study population. Spain et al. found that irTCP was more common in combination ICI approaches; our study confirms this finding, but we were unable to identify any other reliable risk factors [37].

The literature regarding irTCP and OS is sparse, and our finding that irTCP may be associated with worse OS goes against a trend in ICI therapy. Several studies have shown that irAEs are associated with improved OS [4, 23, 38–44]. Delanoy et al. reviewed 948 patients from three French pharmacovigilance databases and identified thirty-five with hematologic irAEs probably or certainly related to anti-PD-1 or anti-PD-L1 immunotherapy; the most commonly identified were neutropenia, autoimmune hemolytic anemia, and immune thrombocytopenia in nine patients (26%) each [45]. A total of twelve out of the thirty-five patients died following hematologic irAE within the follow-up period, two of which were attributed as fatal irAEs.

Our study shows an association between grade 3 and 4 immune-related thrombocytopenia and worse overall survival. One possible explanation for this difference is that those with irTCP have increased risk of clinically significant bleeding events which may either directly lead to death or to stopping of ICI. In this study, four of the eighteen cases with irTCP had bleeding complications and one was the cause of death. Further studies should explore whether the risk of clinically significant bleeding events is different among those with irTCP versus those with other causes of thrombocytopenia. Additionally, given that based on one good observed outcome, eltrombopag should be considered further as a possible treatment for irTCP.

As previously discussed, irTCP may be particularly difficult to identify in this patient population, which may also lead to delay in the appropriate immunosuppressive therapy and thus reversal of significant thrombocytopenia. With ICI now being used in combination with other therapies that may impact the bone marrow such as chemotherapy and radiation, it is important to develop strategies to identify irTCP since the treatment is different than for thrombocytopenia caused by other therapies. In our study, other than type of ICI therapy, we were unable to identify risk factors for the development of irTCP.

This study has several limitations. Although it is a large sample, it is predominantly made up of those treated with anti-PD-1/PD-L1 therapies at a single cancer center. The sample also has ICIs used in the first, second, and third line of therapy and it would be useful to have a more homogenous patient population to explore whether findings are similar. Future studies are needed to identify risk factors for irTCP especially as the use of ICI in combination with conventional chemotherapy becomes more common and therefore attribution of toxicity even more challenging.

### Conclusions

Immune-related thrombocytopenia is a rare irAE with an incidence of 1.73% in this study, which is in line with previous estimates. irTCP was associated with worse overall survival in patients treated with ICI. These findings must be confirmed in larger prospective studies and should include the new landscape of ICI therapy.

### Supplementary Information

Below is the link to the electronic supplementary material.

Supplementary file1 (DOCX 20 kb)
