# Supplementary material for: Immune checkpoint inhibitor-related thrombocytopenia: incidence, risk factors and effect on survival
Source: Cancer Immunol Immunother. 2021 Oct 7;71(5):1157–65. doi: 10.1007/s00262-021-03068-2 (PMC9015999; doi:10.1007/s00262-021-03068-2)
Supplement: Supplementary file 1 — Supplementary file1 (DOCX 20 kb) [file 262_2021_3068_MOESM1_ESM.docx]

**Supplementary Table 1** Patient details for those who developed immune-related thrombocytopenia.

| **Case** | **Cancer type** | **Immunotherapy** | **C1D1 Plt/mcL (grade)** | **Nadir Plt/mcL (grade)** | **Days to nadir** | **Cause of death** |
| --- | --- | --- | --- | --- | --- | --- |
| A | Melanoma | Ipilumumab/nivolumab | 106000 (1) | 20000 (4) | 113 | PD |
| B | Ovarian | Pembrolizumab | 165000 (0) | 3000 (4) | 93 | AHRF |
| C | Urothelial carcinoma | Atezolizumab | 200000 (0) | 27000 (3) | 238 | GIB |
| D | Melanoma | Ipilumumab | 185000 (0) | 12000 (4) | 33 | PD |
| E | Head and neck | Pembrolizumab | 125000 (1) | 13000 (4) | 58 | PD |
| F | DLBCL | Pembrolizumab | 117000 (1) | 13000 (4) | 55 | PD |
| G | Melanoma | Ipilumumab | 363000 (0) | 14000 (4) | 28 | PD |
| H | Melanoma | Ipilumumab/nivolumab | 59000 (2) | 16000 (4) | 71 | Alive |
| I | Melanoma | Ipilumumab/nivolumab | 251000 (0) | 17000 (4) | 72 | PD |
| J | Head and neck | Pembrolizumab | 104000 (1) | 18000 (4) | 243 | PD |
| K | DLBCL | Pembrolizumab | 140000 (1) | 19000 (4) | 138 | PD |
| L | NSCLC | Pembrolizumab | 202000 (0) | 23000 (4) | 81 | PD |
| M | Urothelial carcinoma | Nivolumab | 177000 (0) | 25000 (3) | 131 | PD |
| N | Melanoma | Ipilumumab/nivolumab | 222000 (0) | 27000 (3) | 57 | Sepsis |
| O | Melanoma | Nivolumab | 67000 (2) | 31000 (3) | 29 | PD |
| P | DLBCL | Pembrolizumab | 239000 (0) | 44000 (3) | 28 | PD |
| Q | Melanoma | Ipilumumab/nivolumab | 160000 (0) | 47000 (3) | 6 | PD |
| R | Melanoma | Pembrolizumab | 77000 (2) | 47000 (3) | 14 | PD |

(DLBCL) diffuse large B cell lymphoma, (PD) disease progression, (AHRF) acute hypoxic respiratory failure, (GIB) Gastrointestinal bleed.
